# Accurate Determination of the Bandgap Energy of the Rare-Earth Niobate Series

**DOI:** 10.1021/acs.jpclett.3c00020

**Published:** 2023-02-10

**Authors:** Alka B. Garg, David Vie, Placida Rodriguez-Hernandez, Alfonso Muñoz, Alfredo Segura, Daniel Errandonea

**Affiliations:** † High Pressure and Synchrotron Radiation Physics Division, Bhabha Atomic Research Centre, Mumbai 400085, India; ‡ Homi Bhabha National Institute, Anushaktinagar, Mumbai 400094, India; § Institut de Ciència dels Materials de la Universitat de València, Apartado de Correos 2085, E-46071 València, Spain; ∥ Departamento de Física, Instituto de Materiales y Nanotecnología, MALTA Consolider Team, Universidad de La Laguna, La Laguna, E-38204 Tenerife, Spain; ⊥ Departamento de Física Aplicada-ICMUV, MALTA Consolider Team, Universidad de Valencia, Edificio de Investigación, Carrer del Dr. Moliner 50, Burjassot, 46100 Valencia, Spain

## Abstract

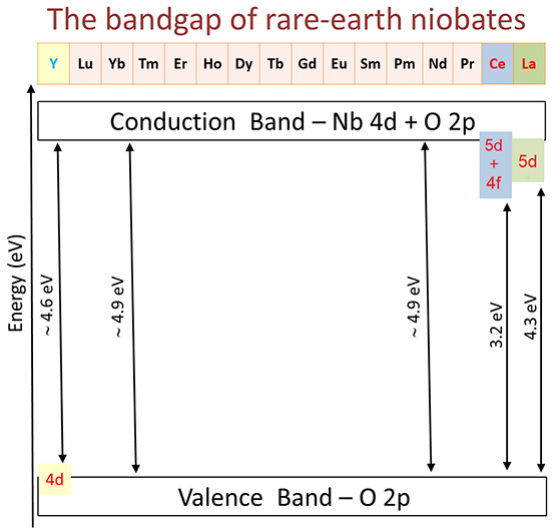

We report diffuse reflectivity measurements in InNbO_4_, ScNbO_4_, YNbO_4_, and eight rare-earth niobates. A comparison with established values of the bandgap of InNbO_4_ and ScNbO_4_ shows that Tauc plot analysis gives erroneous estimates of the bandgap energy. Conversely, accurate results are obtained considering excitonic contributions using the Elliot–Toyozawa model. The bandgaps are 3.25 eV for CeNbO_4_, 4.35 eV for LaNbO_4_, 4.5 eV for YNbO_4_, and 4.73–4.93 eV for SmNbO_4_, EuNbO_4_, GdNbO_4_, DyNbO_4_, HoNbO_4_, and YbNbO_4_. The fact that the bandgap energy is affected little by the rare-earth substitution from SmNbO_4_ to YbNbO_4_ and the fact that they have the largest bandgap are a consequence of the fact that the band structure near the Fermi level originates mainly from Nb 4d and O 2p orbitals. YNbO_4_, CeVO_4_, and LaNbO_4_ have smaller bandgaps because of the contribution from rare-earth atom 4d, 5d, or 4f orbitals to the states near the Fermi level.

Rare-earth and trivalent metals niobates (MNbO_4_) have been used for half a century as luminescent materials.^1^ These compounds crystallize in the monoclinic fergusonite structure (space group *I*2/*a* also described as *I*2/*b* or *C*2/*c* depending on the choice of vectors used to define the unit cell) and have exceptional chemical stability, luminescent characteristics, dielectric properties, and ionic conductivities.^2^ Because of this, they have been proposed for many different applications. These include lasers, light emitters, capacitors, optical fibers, medical applications, temperature detectors, bioimaging, photocatalysis for both contaminant degeneration and H_2_ generation, chemically robust hosts for nuclear materials and wastes, ion conductors for lithium batteries, solid oxide fuel cells, etc.^2−6^ The fact that MNbO_4_ niobates can be obtained as single crystals^7^ and nanocrystals^8^ leads to a great versatility regarding applications. One of the applications of MNbO_4_ niobates that has been gaining momentum in the past several years is their use for environmentally friendly white-light-emitting diodes (LEDs).^9,10^ In the past several years, these LEDs have replaced conventional light sources, including Edison’s incandescent lamp and the Hg discharge-based fluorescent lamp. LEDs have many advantages over the other light sources, including lower power consumption, longer lifetime, improved physical robustness, smaller size, and faster switching.^11^ One of the crucial steps to further optimize white LEDs based on orthoniobates is the precise determination of the energy of their electronic bandgap (*E*_gap_), which is still lacking. Experimental and density functional theory efforts have been devoted to it, but the information in the literature^2,4,5,12−16^ is limited to only some of the rare-earth niobates and, in some cases, contradictory as one can see in Table 1. Notice that bandgap values from 2.9 to 5.04 eV have been reported for orthoniobates. Such a large variation in *E*_gap_ contradicts the know-systematic of related orthovanadates, which have been extensively studied.^17,18^ In particular, in YNbO_4_ (*E*_gap_ = 3.7–4.96 eV), LuNbO_4_ (*E*_gap_ = 4.2–5.04 eV), and GdNbO_4_ (*E*_gap_ = 3.48–4.89 eV), there are significant discrepancies in the literature. These facts indicate that a systematic study of the bandgap energy of MNbO_4_ niobates is timely. Here we report diffuse reflectance measurements in YNbO_4_, LaNbO_4_, CeNbO_4_, SmNbO_4_, EuNbO_4_, GdNbO_4_, DyNbO_4_, HoNbO_4_, and YbNbO_4_. A systematic analysis of the results using an Elliot–Toyozawa model^19,20^ has allowed us to accurately determine the bandgap energy of the studied compounds. This analysis reveals that the traditional method of determination based on the Tauc plot^21^ underestimates the bandgap energy. Our method for the determination of *E*_gap_ has been validated by performing diffuse reflectance measurements in InNbO_4_ and ScNbO_4_, giving an excellent agreement with accurate bandgap values determined via luminescence measurements, 4.7 and 4.8 eV, respectively.^22,23^ From our study, we conclude that most rare-earth niobates have a bandgap energy of 4.73–4.93 eV, with only CeNbO_4_ (3.25 eV), LaNbO_4_ (4.35 eV), and YNbO_4_ (4.55 eV) having narrower bandgaps. The observed results will be explained using available band-structure calculations.

**Table 1:** Bandgap Energies of Orthoniobates in Electronvolts^a^

compound	Elliot fit	Tauc plot	literature value(s)	refs
InNbO_4_	4.7	4.35	4.7	(22)
ScNbO_4_	4.8	4.38	4.8	(23)
YNbO_4_	4.55	4.15	3.7–4.96	(2), (4), (5), (14)
LaNbO_4_	4.35	3.80	4.0	(13)
CeNbO_4_	3.25	2.65		
PrNbO_4_			4.8	(15)
NdNbO_4_				
SmNbO_4_	4.95	4.45	4.7–5.0	(33)
EuNbO_4_	4.73	4.30	3.45	(12)
GdNbO_4_	4.93	4.50	3.48–4.89	(4), (12)
TbNbO_4_			2.9	(5)
DyNbO_4_	4.93	4.55		
HoNbO_4_	4.93	4.55		
ErNbO_4_			3.5	(16)
TmNbO_4_				
YbNbO_4_	4.90	4.45	3.46	(16)
LuNbO_4_			4.2–5.04	(2), (4)

a Results obtained using the Elliot model and the Tauc plot are compared with values from the literature.

We start by presenting the results for InNbO_4_ and ScNbO_4_, which have been used to establish the method used to determine *E*_gap_. Both materials have a monoclinic wolframite-type structure (related to fergusonite), and their bandgap energies have been accurately determined previously.^22,23^ In Figure 1, we present the absorption spectra *F*(*R*_∞_) obtained from the diffuse reflectance measurements via the Kubelka–Munk transformation,^24^ which can be considered approximately proportional to the absorption coefficient (α).^25^ In both InNbO_4_ and ScNbO_4_, the absorbance has a sharp absorption onset above 4 GPa, reaching a maximum at 4.7 and 4.75 eV, respectively (see Figure 1). In the literature,^22,23^ there is an agreement that InNbO_4_ and ScNbO_4_ have direct bandgaps with *E*_gap_ values of 4.7 and 4.8 eV, respectively. However, if we analyze our results using the traditional Tauc plot analysis,^21^ determining *E*_gap_ from a linear least-squares fit to zero in [*hυF*(*R*_∞_)]^2^ versus *hυ* (as shown in Figure 1), we find values for *E*_gap_ that are 0.4 eV smaller than the consensus values for *E*_gap_ in InNbO_4_ and ScNbO_4_.^22,33^ We are not surprised by this fact because this method has been recently challenged.^26^ The main drawback is that it tends to underestimate the bandgap energy in materials due to sub-bandgap absorption tails related to defects, surface effects, and other phenomena that are reflected in the absorption spectrum as an Urbach tail.^26^ The fact that the Tauc analysis neglects the presence of excitons could also lead to the underestimation of *E*_gap_.^27^ In materials related to MNbO_4_ niobates, like InVO_4_, the application of the traditional Tauc plot analysis leads to underestimations of ≤1.8 eV in the value of *E*_gap_.^28^ Then, it is thus not surprising that in InNbO_4_ and ScNbO_4_, it could lead to an underestimation of 0.4 eV. We will show now that this is because of the excitonic contribution to the fundamental absorption spectrum. In InNbO_4_ and ScNbO_4_, the steplike absorption present above 4 eV (see Figure 1) is typical of direct transitions in which excitonic effects are observed even at room temperature.^29^ This can be confirmed by fitting the absorbance obtained by means of the excitonic Elliott–Toyozawa model^19,20^ in which
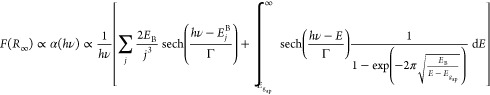
In this expression, the first term within the brackets is the discrete excitonic contribution and the second term is the continuum contribution. In our fitting, we have assumed that only the fundamental exciton state (*j* = 1) contributes to the peak, being *E*_1_^B^ = *E*_gap_ – *E*_B_, where *E*_B_ is the exciton binding energy. The only fitting parameters are *E*_gap_, *E*_B_, and Γ, which take the spectral line width into account. This simple model can reproduce the absorbance of the two compounds as shown in Figure 1. From our fit, we have determined *E*_gap_ values of 4.7 for InNbO_4_ and 4.8 eV for ScNbO_4_. These values are in excellent agreement with the literature.^22,23^ The obtained *E*_B_ values are 70 and 120 meV, respectively. These values are comparable to the exciton binding energy of wide bandgap oxides.^29^ The excellent agreement with the literature and the good quality of fits (using a simple model with only the fundamental exciton) confirm that excitonic contributions are important in the absorbance of MNbO_4_ niobates. These contributions are caused by interactions between electrons and holes that produce extended states that cause a narrowing of the bandgap.^19,20^ We consequently will use the same model to analyze all of the rare-earth niobates we have investigated.

**Figure 1:**
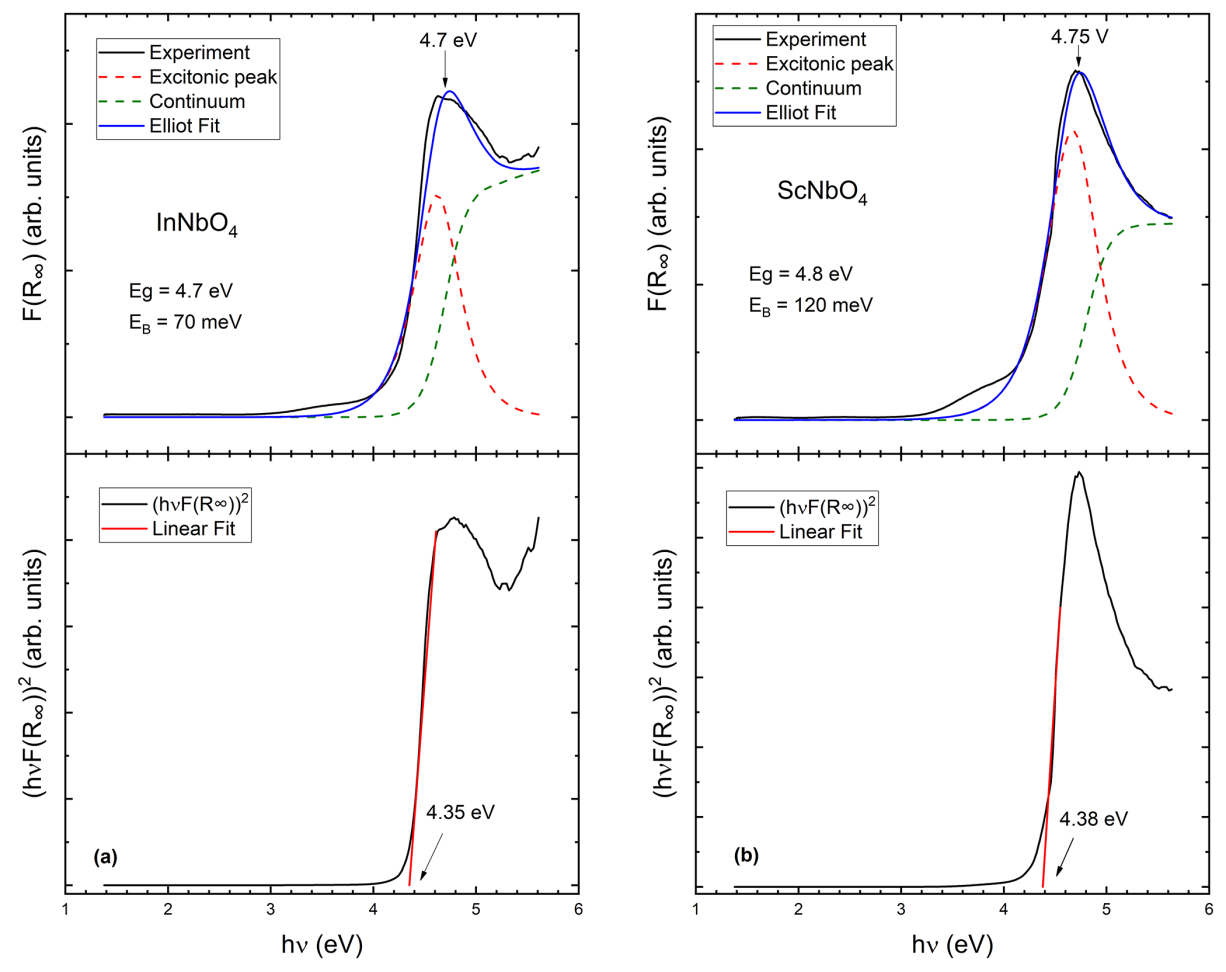
(a) *F*(*R*_∞_) spectra of InNbO_4_ together with the fit used to determine the bandgap energy. The black line is the experiment, the blue line the fit, the green dashed line the continuum contribution, and the red dashed line the excitonic contribution. The arrow shows the maximum of the abosrbance. The bottom plot shows the Tauc plot traditionally used to determine *E*_gap_. (b) Same arrangement but for ScNbO_4_.

Once the correct method for determining the bandgap energy has been established, we will present the results for rare-earth niobates. We will first present the results for SmNbO_4_, EuNbO_4_, and GdNbO_4_. The results of our experiments and the analysis are provided in Figure 2. The three compounds have a sharp absorption edge typical of a direct bandgap. Below that energy, there is a sub-bandgap Urbach tail, typical of MXO_4_^30^ oxides and weak peaks that can be attributed to internal 4f–4f transitions associated with the lanthanide cation.^31,32^ Upon application of the Elliott–Toyozawa model, the fundamental absorption edge can be fitted quite well. The determined bandgap energies are 4.95, 4.73, and 4.93 eV for SmNbO_4_, EuNbO_4_, and GdNbO_4_, respectively. The obtained values for the exciton binding energy (see Figure 2) are comparable to the values determined for InNbO_4_ and ScNbO_4_. The bandgap energy is very close to the energy of the maximum of *F*(*R*_∞_) as shown in Figure 2. In the figure, we also include a fit using the Tauc plot. The values estimated for *E*_gap_ with this method are always <0.4 eV compared to the correct value as one can see in Table 1. In this table, we also compare our results with those of previous studies. For SmNbO_4_, this is the first determination of *E*_gap_. The value obtained is consistent with photoluminescence measurements, which constrain the bandgap energy to 4.7–5.0 eV.^33^ For the case of GdNbO_4_, our result (4.93 eV) is in excellent agreement with the result of Feng et al. (4.89 eV).^12^ This indicates that the bandgap of 3.48 eV reported by Hirano et al. is an underestimation. The same can be stated for the bandgap of 3.45 eV determined by the same authors for EuNbO_4_,^12^ which is 1.3 eV smaller than the value of *E*_gap_ determined in the work presented here.

**Figure 2:**
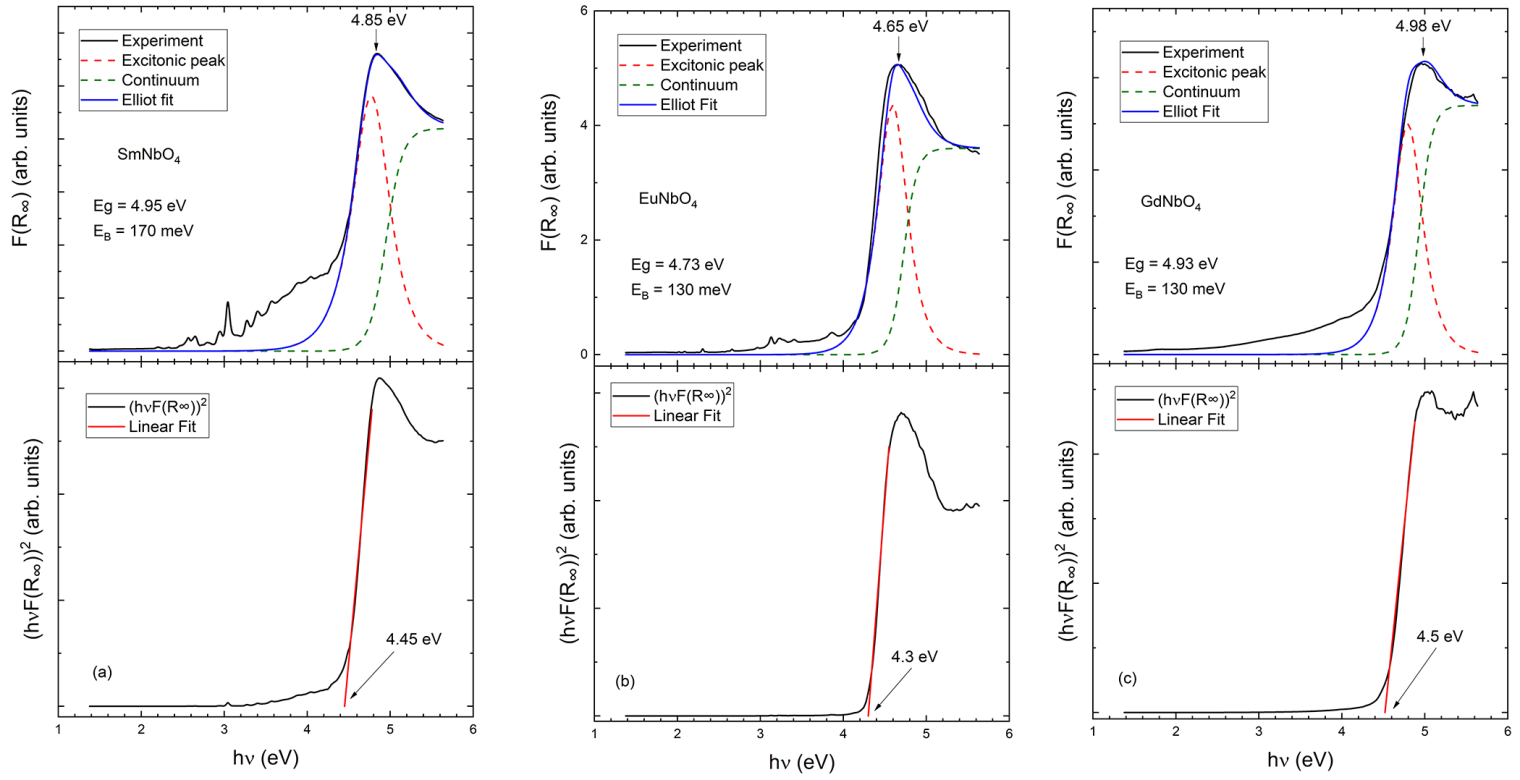
(a) *F*(*R*_∞_) spectra of SmNbO_4_ (top) together with the fit used to determine the bandgap energy. The black line is the experiment, the blue line the fit, the green dashed line the continuum contribution, and the red dashed line the excitonic contribution. The arrow shows the maximum of the abosrbance. The bottom plot shows the Tauc plot traditionally used to determine *E*_gap_. (b) Same arrangement but for EuNbO_4_. (c) Same arrangement but for GdNbO_4_.

Figure 3 shows the results obtained for DyNbO_4_, HoNbO_4_, and YbNbO_4_. Again, the three compounds have a very large bandgap (*E*_gap_) of ∼4.9 eV (see Figure 3 and Table 1) comparable to that of the widest bandgap semiconductors, for instance, Ga_2_O_3_.^29,34^Figure 3 shows that the bandgap energy is very close to the energy of the maximum of *F*(*R*_∞_). The figure also shows how *E*_gap_ is underestimated by ∼0.4 eV when Tauc plot analysis is performed. Notice that *E*_gap_ in the three compounds is very similar to *E*_gap_ in SmNbO_4_, EuNbO_4_, and GdNbO_4_. As we will explain toward the end of this work, this is a consequence of the fact that the electronic states at the bottom of the conduction band and the top of the valence band are dominated by O 2p states and Nb 4d states. Thus, as a first approximation, the bandgap is determined by the configuration of the NbO_4_ tetrahedron, which does not change significantly from one compound to the other. The three of them also have the typical Urbach tail and a contribution from absorptions from the 4f levels of the rare-earth atoms. In addition, HoNbO_4_ and DyNbO_4_ have in the *F*(*R*_∞_) sub-bandgap sharp absorptions caused by internal 4f–4f transitions of Dy and Ho.^32^ For DyNbO_4_ and HoNbO_4_, this is the first time that the band energy has been reported. In YbNbO_4_, our result (*E*_gap_ = 4.9 eV) shows that *E*_gap_ has been largely underestimated in a previous report.^16^

**Figure 3:**
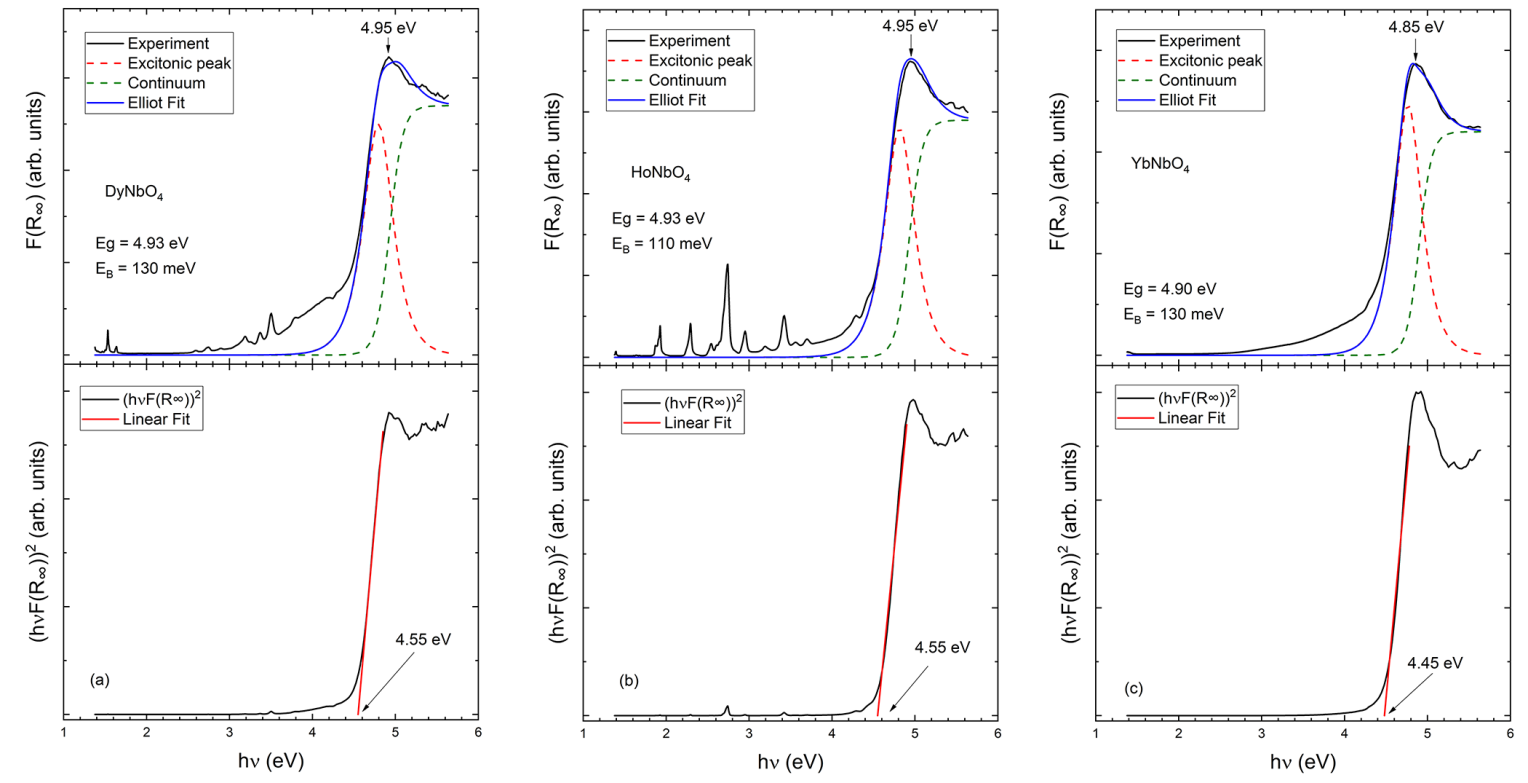
(a) *F*(*R*_∞_) spectra of DyNbO_4_ (top) together with the fit used to determine the bandgap energy. The black line is the experiment, the blue line the fit, the green dashed line the continuum contribution, and the red dashed line is the excitonic contribution. The arrow shows the maximum of the abosrbance. The bottom plot shows the Tauc plot traditionally used to determine *E*_gap_. (b) Same arrangement but for HoNbO_4_. (c) Same arrangement but for YbNbO_4_.

In Figure 4, we present the results obtained for YNbO_4_, LaNbO_4_, and CeNbO_4_. The figure shows that Tauc analysis always underestimates values of *E*_gap_. According to the Elliot–Toyozawa model, YNbO_4_ has a bandgap energy of 4.56 eV (see Figure 4 and Table 1). This value is 10% smaller than in the previously discussed niobates, and it is comparable to the maximum value of *E*_gap_ reported in previous studies.^2,4,5,14^ For LaNbO_4_, we have determined an *E*_gap_ of 4.35 eV. The previously determined value was 10% smaller.^13^ For CeNbO_4_, this is the first time that *E*_gap_ has been determined. The obtained value, 3.25 eV, indicates that this compound is the orthoniobate with the smallest bandgap. As in the other studied compounds, in the three compounds presented in Figure 4, the bandgap energy is very close to the energy of the maximum of *F*(*R*_∞_).

**Figure 4:**
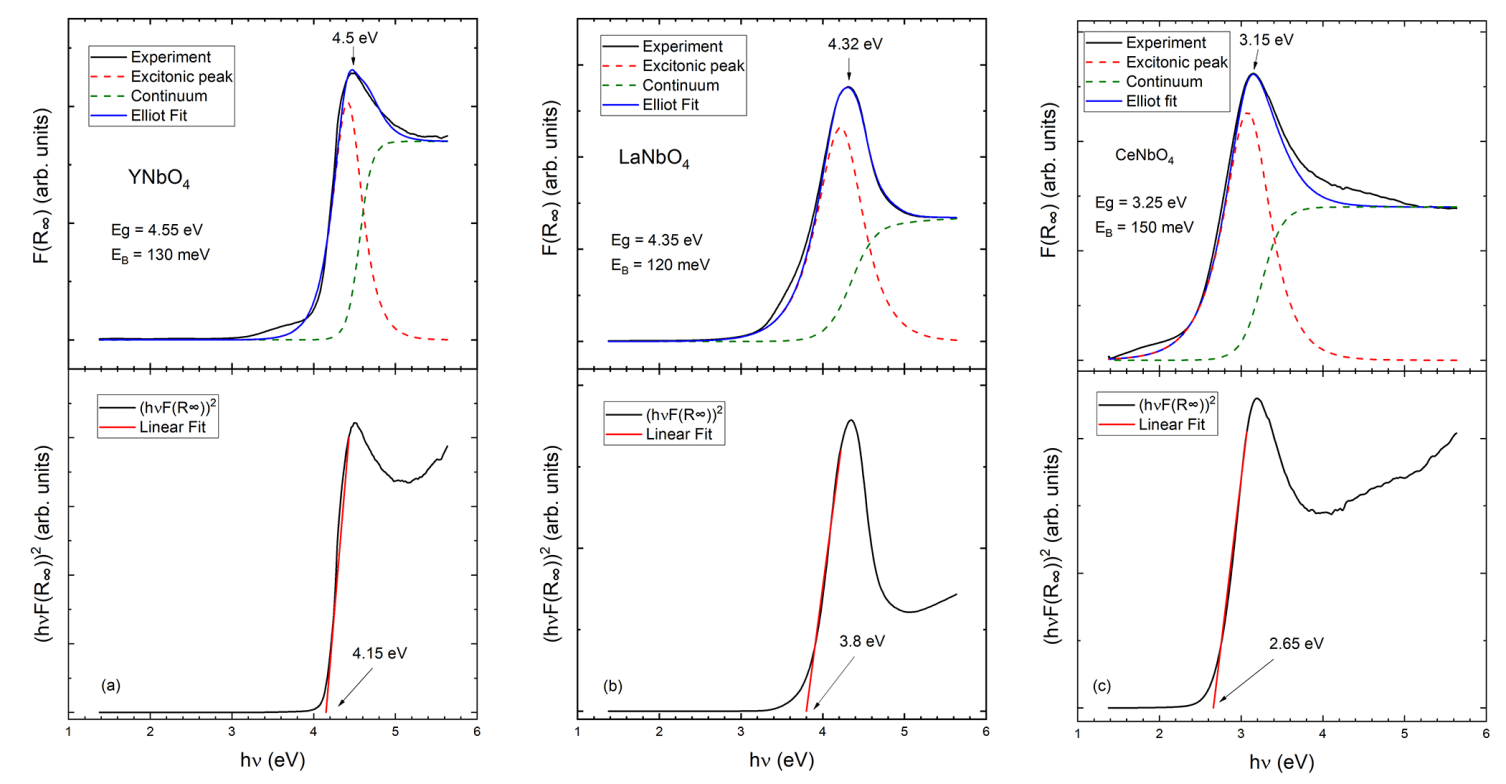
(a) *F*(*R*_∞_) spectra of YNbO_4_ (top) together with the fit used to determine the bandgap energy. The black line is the experiment, the blue line the fit, the green dashed line the continuum contribution, and the red dashed line the excitonic contribution. The arrow shows the maximum of the abosrbance. The bottom plot shows the Tauc plot traditionally used to determine *E*_gap_. (b) Same arrangement but for LaNbO_4_. (c) Same arrangement but for CeNbO_4_.

It is interesting to stress that the bandgap energy of rare-earth niobates follows a very similar trend as observed for rare-earth vanadates.^17,35,36^ In the vanadates, except for LaVO_4_ and CeVO_4_, all of the members of the family have a bandgap close to 3.8 eV. LaVO_4_ has a bandgap of 3.5 eV, and CeVO_4_ a bandgap of 3.2 eV. The 3.8 eV bandgap is a consequence of the fact that states near the Fermi level are dominated by O 2 p and V 3 d states. In the other compounds, the bandgap is reduced due to the contribution of 5d or 4f electrons from La and Ce. A similar picture provides a qualitative explanation for the results reported here. The band structure and electronic density of states (DOS) of most niobates have been reported in the literature.^2,13,37−39^ They are qualitatively similar to the band structure and DOS of vanadates.^35^ In particular, the band structures of niobates and vanadates have a similar topology. The states near the Fermi level in niobates have a composition qualitatively similar to that in vanadates, being dominated by O 2p and Nb 4d (instead of V 3d) states. According to band-structure calculations, the studied niobates have an indirect gap, but the difference with the direct bandgap is <0.1 eV.^2,13,37−39^ Then the absorption edge will be dominated by the direct bandgap and *F*(*R*_∞_) can be analyzed assuming a direct bandgap as it has been done in this work.

To provide a more quantitative discussion, we will focus on LaNbO_4_, CeNbO_4_, EuNbO_4_, and YbNbO_4_,^38^ which are representative of the family of fergusonite-type niobates. In the four compounds, the minimum of the conduction band (CB) and the maximum of the valence band (VB) are located at the V and Γ points, respectively, of the Brillouin zone. However, there is a Γ–Γ direct bandgap very close in energy. According to the electronic DOS, in EuNbO_4_ and YbNbO_4_ the valence-band maximum is dominated by O 2p states. This behavior is typical of most wide bandgap oxides. On the contrary, the conduction-band minimum is predominantly dominated by Nb 4d states with a small contribution of O 2p states. This molecular orbital composition is analogous to what happens in vanadates with V 3d and O 2p states^35^ and in other ternary oxides like tungstates (molybdates) with W (Mo) 5d (4d) and O 2p states.^40^ Consequently, the configuration of the NbO_4_ tetrahedron, the coordination polyhedron of Nb in fergusonite, will have a determining role in the evaluation of the bandgap energy. Interestingly, the NbO_4_ tetrahedron is not modified significantly in going from one compound to the other, changing the average Nb–O bond distance by <1% along the series of rare-earth niobates. Thus, it is not surprising that most members of this family have a very similar *E*_gap_. It is also not surprising that these compounds have a bandgap energy comparable to that of Nb_2_O_5_ (*E*_gap_ = 5.1 eV) in which states at the bottom of the CB and top the VB are also dominated by the hybridization of O 2p and Nb 4d states, and O 2p states, respectively.^41^ The reason for the bandgap energy closure of LaNbO_4_ and CeNbO_4_ is the fact that these are the only two compounds in which the lanthanide orbitals contribute to the conduction band. This is why LaNbO_4_ and CeNbO_4_ are the niobates with the smallest bandgap within rare-earth niobates. Exactly the same phenomenon happens in LaVO_4_ and CeVO_4_, which are the vanadates with the smallest bandgap energy within the family of rare-earth vanadates.^17^

In summary, herein we report diffuse reflectance measurements for rare-earth niobates and propose a method for accurately determining their bandgap energy. We have shown that in most previous studies the bandgap energy has been underestimated. The bandgap energies of the studied compounds are 3.25 eV for CeNbO_4_, 4.35 eV for LaNbO_4_, 4.5 eV for YNbO_4_, and 4.73–4.93 eV for SmNbO_4_, EuNbO_4_, GdNbO_4_, DyNbO_4_, HoNbO_4_, and YbNbO_4_. We also provide an explanation of the experimental results based on density functional calculations. The reported information is crucial for technological applications of rare-earth niobates. To conclude, we add two comments. (1) If the excitonic peak is not observed, obviously the Elliot–Toyozawa model cannot be accurately used. In such a case, the Tauc method can be used, but keeping in mind that the obtained energy is an energy that is always smaller than real bandgap energy, being sensitive to the thickness of the sample.^29^ (2) If the bandgap is indirect, the contribution of an indirect transition to the absorption coefficient is much weaker than that of an allowed direct transition and can be measured only when the indirect gap is situated at least 0.2–0.3 eV below the direct one.^42^ In such cases, the bandgap energy could be determined assuming for the absorption coefficient a dependence on the square root of the energy: , where *A* is a scaling parameter and *E*_gapind_ is the bandgap energy of the indirect gap.^43^

## Methods

*Experimental Details*. Polycrystalline rare-earth niobates were synthesized by the commonly used solid state reaction technique.^44^ Starting metal oxides, R_2_O_3_ and Nb_2_O_5_ (purity of >99.9%), were predried to remove any moisture or organic impurities. These dried binary oxides were weighed in a stoichiometric (1:1) ratio, hand mixed with a mortar and pestle, cold pressed into cylinders of 12.5 mm in diameter and 5 mm in height, and fired at 1200 °C for 24 h in a box-type programmable resistive furnace. These pellets were further sintered at 1300 °C for 48 h. Single phase formation of the compounds was confirmed by angle-dispersive powder X-ray diffraction. We confirmed that all synthesized rare-earth niobates crystallize in the fergusonite structure. For measurements of ScNbO_4_ and InNbO_4_, we used the same samples used in previous studies.^22,23^ For the diffuse reflectance measurements, powder samples were manually ground in an agate mortar, placed in the sample holder, and supported with a quartz window. Measurements were carried out on a Shimazdu UV–vis 2501PC spectrophotometer equipped with an integrating sphere for diffuse reflectance measurements. BaSO_4_ was used as a reference material and for background measurement. The spectral range covered by measurements was 220–900 nm, with a resolution of 1 nm and a spectral bandwidth of 5 nm. The spectra recorded in %*R* were transformed into absorbance spectra using the Kubelka–Munk function.^24^ The plotted *F*(*R*_∞_) spectra are proportional to the absorption coefficient and can be interpreted with the same physical model.^25^

## References

[ref1] Blasse G.; Bril A. Luminescence Phenomena in Compounds with Fergusonite Structure. J. Lumin. 1970, 3, 109–131. 10.1016/0022-2313(70)90011-6.

[ref2] Ding D.; Zhang H.; Liu W.; Sun D.; Zhang Q. Experimental and First Principle Investigation the Electronic and Optical Properties of YNbO_4_ and LuNbO_4_ Phosphors. J. Mater. Science: Materials in Electronics 2018, 29, 11878–11885. 10.1007/s10854-018-9288-5.

[ref3] Graça M. P. F.; Peixoto M. V.; Ferreira N.; Rodrigues J.; Nico C.; Costa F. M.; Monteiro T. Optical and Dielectric Behaviour of EuNbO_4_ Crystals. J. Mater. Chem. C 2013, 1, 2913–2919. 10.1039/c3tc00793f.

[ref4] Feng Z.; Lou B. B.; Chen Q.; Yin M.; Ma C. G.; Duan C. K. Self-Activated and Bismuth-Related Photoluminescence in Rare-Earth Vanadate, Niobate, and Tantalate Series: A First-Principles Study. Inorg. Chem. 2021, 60, 16614–16625. 10.1021/acs.inorgchem.1c02508. 34648277

[ref5] Hirano M.; Dozono H. Hydrothermal Formation and Characteristics of Rare-Earth Niobate Phosphors and Solid Solutions between YNbO_4_ and TbNbO_4_. Mater. Chem. Phys. 2014, 143, 860–866. 10.1016/j.matchemphys.2013.10.025.

[ref6] Nyman M.; Rodriguez M. A.; Rohwer L. E. S.; Martin J. E.; Waller M.; Osterloh F. E. Unique LaTaO_4_ Polymorph for Multiple Energy Applications. Chem. Mater. 2009, 21, 4731–4737. 10.1021/cm9020645.

[ref7] Fulle K.; McMillen C. D.; Sanjeewa L. D.; Kolis J. W. Hydrothermal Chemistry and Growth of Fergusonite-type RENbO_4_ (RE = La–Lu, Y) Single Crystals and New Niobate Hydroxides. Cryst. Growth Des. 2016, 16, 4910–4917. 10.1021/acs.cgd.6b00466.

[ref8] Hirano M.; Dozono H. Direct Formation and Luminescence Properties of Yttrium Niobate YNbO_4_ Nanocrystals via Hydrothermal Method. J. Am. Ceram. Soc. 2013, 96, 3389–3393. 10.1111/jace.12595.

[ref9] Liu X.; Lü Y.; Chen C.; Luo S.; Zeng Y.; Zhang X.; Shang M.; Li C.; Lin J. Synthesis and Luminescence Properties of YNbO_4_:A (A = Eu^3+^ and/or Tb^3+^) Nanocrystalline Phosphors via a Sol–Gel Process. J. Phys. Chem. C 2014, 118, 27516–27524. 10.1021/jp508773t.

[ref10] Lü Y.; Tang X.; Yan L.; Li K.; Liu X.; Shang M.; Li C.; Lin J. Synthesis and Luminescent Properties of GdNbO_4_:RE^3+^ (RE = Tm, Dy) Nanocrystalline Phosphors via the Sol–Gel Process. J. Phys. Chem. C 2013, 117, 21972–21980. 10.1021/jp4086415.

[ref11] Lin C. C.; Liu R. S. Advances in Phosphors for Light-emitting Diodes. J. Phys. Chem. Lett. 2011, 2, 1268–1277. 10.1021/jz2002452. 26295420

[ref12] Hirano M.; Ishikawa K. Direct Synthesis of Nanocrystalline GdNbO_4_ and GdNbO_4_-Based Phosphors Doped with Eu^3+^ through Hydrothermal Route. J. Cer. Soc. Japan 2016, 124, 42–48. 10.2109/jcersj2.15193.

[ref13] Arai M.; Wang Y. X.; Kohiki S.; Matsuo M.; Shimooka H.; Shishido T.; Oku M. Dielectric Property and Electronic Structure of LaNbO_4_. Jpn. J. Appl. Phys. 2005, 44, 6596–6599. 10.1143/JJAP.44.6596.

[ref14] Lee S. K.; Chang H.; Han C. H.; Kim H. J.; Jang H. G.; Park H. D. Electronic Structures and Luminescence Properties of YNbO_4_ and YNbO_4_:Bi. J. Solid State Chem. 2001, 156, 267–273. 10.1006/jssc.2000.8941.

[ref15] Peixoto J. C.; Dias A.; Matinaga F. M.; Siqueira K. P. F. Luminescence Properties of PrNbO_4_ and EuNbO_4_ Orthoniobates and Investigation of Their Structural Phase Transition by High-Temperature Raman Spectroscopy. J. Lumin. 2021, 238, 118284 10.1016/j.jlumin.2021.118284.

[ref16] Zhang Z.; Guo L.; Sun H.; Peng D.; Zou H.; Sun N.; Zhang Q.; Hao X. Rare Earth Orthoniobate Photochromics with Self-Activated Upconversion Emissions for High-Performance Optical Storage Applications. J. Mater. Chem. C 2021, 9, 13841–13850. 10.1039/D1TC02987H.

[ref17] Errandonea D. High Pressure Crystal Structures of Orthovanadates and Their Properties. J. Appl. Phys. 2020, 128, 040903 10.1063/5.0016323.

[ref18] Errandonea D.; Garg A. B. Recent Progress on the Characterization of the High-Pressure Behaviour of AVO_4_ Orthovanadates. Prog. Mater. Science 2018, 97, 123–169. 10.1016/j.pmatsci.2018.04.004.

[ref19] Elliott R. J. Intensity of Optical Absorption by Excitons. Phys. Rev. 1957, 108, 1384–1389. 10.1103/PhysRev.108.1384.

[ref20] Toyozawa Y. Theory of Line-Shapes of the Exciton Absorption Bands. Prog. Theor. Phys. 1958, 20, 53–81. 10.1143/PTP.20.53.

[ref21] Tauc J. Optical Properties and Electronic Structure of Amorphous Ge and Si. Mater. Res. Bul. 1968, 3, 37–46. 10.1016/0025-5408(68)90023-8.

[ref22] Botella P.; Enrichi F.; Vomiero A.; Muñoz-Santiuste J. E.; Garg A. B.; Arvind A.; Manjón F. J.; Segura A.; Errandonea D. Investigation on the Luminescence Properties of InMO_4_ (M = V^5+^, Nb^5+^, Ta^5+^) Crystals Doped with Tb^3+^ or Yb^3+^ Rare Earth Ions. ACS Omega 2020, 5, 2148–2158. 10.1021/acsomega.9b02862. 32064375 PMC7016905

[ref23] Ouahrani T.; Garg A. B.; Rao R.; Rodríguez-Hernández P.; Muñoz A.; Badawi M.; Errandonea D. On the High-Pressure Properties of Wolframite-type ScNbO_4_. J. Phys. Chem. C 2022, 126, 4664–4676. 10.1021/acs.jpcc.1c10483.

[ref24] Kubelka P.; Munk F. An Article on Optics of Paint Layers. Z. Technol. Phys. 1931, 12, 593–603.

[ref25] Dolgonos A.; Mason T. O.; Poeppelmeier K. R. Direct Optical Bandgap Measurement in Polycrystalline Semiconductors: A Critical Look at the Tauc Method. J. Solid State Chem. 2016, 240, 43–48. 10.1016/j.jssc.2016.05.010.

[ref26] Makuła P.; Pacia M.; Macyk W. How to Correctly Determine the Bandgap Energy of Modified Semiconductor Photocatalysts Based on UV–Vis Spectra. J. Phys. Chem. Lett. 2018, 9, 6814–6817. 10.1021/acs.jpclett.8b02892. 30990726

[ref27] Ruf F.; Aygüler M. F.; Giesbrecht N.; Rendenbach B.; Magin A.; Docampo P.; Kalt H.; Hetterich M. Temperature-Dependent Studies of Exciton Binding Energy and Phase-Transition Suppression in (Cs,FA,MA)Pb(I,Br)_3_ Perovskites. APL Mater. 2019, 7, 031113 10.1063/1.5083792.

[ref28] Botella P.; Errandonea D.; Garg A. B.; Rodríguez-Hernández P.; Muñoz A.; Achary S. N.; Vomiero A. High-Pressure Characterization of the Optical and Electronic Properties of InVO_4_, InNbO_4_, and InTaO_4_. SN Appl. Sci. 2019, 1, 389 10.1007/s42452-019-0406-7.

[ref29] Segura A.; Artús L.; Cuscó R.; Goldhahn R.; Feneberg M. Bandgap of Corundum-like α–Ga_2_O_3_ Determined by Absorption and Ellipsometry. Phys. Rev. Mater. 2017, 1, 024604 10.1103/PhysRevMaterials.1.024604.

[ref30] Errandonea D.; Muñoz A.; Rodríguez-Hernández P.; Proctor J. E.; Sapiña F.; Bettinelli M. Theoretical and Experimental Study of the Crystal Structures, Lattice Vibrations, and Band Structures of Monazite-Type PbCrO_4_, PbSeO_4_, SrCrO_4_, and SrSeO_4_. Inorg. Chem. 2015, 54, 7524–7535. 10.1021/acs.inorgchem.5b01135. 26161677

[ref31] Carnall W. T.; Crosswhite H.; Crosswhite H. M. Energy Level Structure and Transition Probabilities in the Spectra of the Trivalent Lanthanides in LaF_3_. Argonne National Laboratory: Argonne, IL, 1978.

[ref32] Bandiello E.; Errandonea D.; Piccinelli F.; Bettinelli M.; Díaz-Anichtchenko D.; Popescu C. Characterization of Flux-Grown Sm_x_Nd_1–x_VO_4_ Compounds and High-Pressure Behavior for x = 0.5. J. Phys. Chem. C 2019, 123, 30732–30745. 10.1021/acs.jpcc.9b09473.

[ref33] Nico C.; Soares M. R. N.; Costa F. M.; Monteiro T.; Graça M. P. F. Structural, Optical, and Electrical Properties of SmNbO_4_. J. Appl. Phys. 2016, 120, 051708 10.1063/1.4958953.

[ref34] Chi Z.; Asher J. J.; Jennings M. R.; Chikoidze E.; Pérez-Tomás A. Ga_2_O_3_ and Related Ultra-Wide Bandgap Power Semiconductor Oxides: New Energy Electronics Solutions for CO_2_ Emission Mitigation. Materials 2022, 15, 1164 10.3390/ma15031164. 35161108 PMC8838167

[ref35] Panchal V.; Errandonea D.; Segura A.; Rodríguez-Hernández P.; Muñoz A.; Lopez-Moreno S.; Bettinelli M. The Electronic Structure of Zircon-Type Orthovanadates: Effects of High-Pressure and Cation Substitution. J. Appl. Phys. 2011, 110, 043723 10.1063/1.3626060.

[ref36] Muñoz-Santiuste J. E.; Lavín V.; Rodríguez-Mendoza U. R.; Ferrer-Roca Ch.; Errandonea D.; Martínez-García D.; Rodríguez-Hernández P.; Muñoz A.; Bettinelli M. Experimental and Theoretical Study on the Optical Properties of LaVO_4_ Crystals under Pressure. Phys. Chem. Chem. Phys. 2018, 20, 27314–27328. 10.1039/C8CP04701D. 30357167

[ref37] Ding S.; Zhang H.; Zhang Q.; Chen Y.; Dou R.; Peng F.; Liu W.; Sun D. Experimental and First Principle Study of the Structure, Electronic, Optical, and Luminescence Properties of M-type GdNbO_4_ Phosphor. J. Sol. State Chem. 2018, 262, 87–93. 10.1016/j.jssc.2018.03.011.

[ref38] Litimein F.; Khenata R.; Gupta S. K.; Murtaza G.; Reshak A. H.; Bouhemadou A.; Bin Omran S.; Yousaf M.; Jha P. K. Structural, Electronic, and Optical Properties of Orthorhombic and Triclinic BiNbO_4_ Determined via DFT Calculations. J. Mater. Sci. 2014, 49, 7809–7818. 10.1007/s10853-014-8491-x.

[ref39] Materials Project web site. https://materialsproject.org/.

[ref40] Liang A.; Turnbull R.; Rodríguez-Hernandez P.; Muñoz A.; Jasmin M.; Shi L. T.; Errandonea D. General Relationship Between the Bandgap Energy and Iodine-Oxygen Bond Distance in Metal Iodates. Phys. Rev. Materials 2022, 6, 044603 10.1103/PhysRevMaterials.6.044603.

[ref41] Sathasivam S.; Williamson B. A. D.; Althabaiti S. A.; Obaid A. Y.; Basahel S. N.; Mokhtar M.; Scanlon D. O.; Carmalt C. J.; Parkin I. P. Chemical Vapor Deposition Synthesis and Optical Properties of Nb_2_O_5_ Thin Films with Hybrid Functional Theoretical Insight into the Band Structure and Bandgaps. ACS Appl. Mater. Interfaces 2017, 9, 18031–18038. 10.1021/acsami.7b00907. 28492079

[ref42] Pellicer-Porres J.; Manjon F. J.; Segura A.; Muñoz V.; Power C.; Gonzalez J. Optical Absorption in GaTe under High Pressure. Phys. Rev. B 1999, 60, 8871–8877. 10.1103/PhysRevB.60.8871.

[ref43] Manjón F.-J.; Errandonea D.; Segura A.; Muñoz V.; Tobías G.; Ordejón P.; Canadell E. Experimental and Theoretical Study of Band Structure of InSe and In. Phys. Rev. B 2001, 63, 125330.

[ref44] Garg A. B.; Rao R.; Errandonea D.; Pellicer-Porres J.; Martinez-Garcia D.; Popescu C. Pressure-Induced Instability of the Fergusonite Phase of EuNbO_4_ Studied by In Situ Raman Spectroscopy, X-ray Diffraction, and Photoluminescence Spectroscopy. J. Appl. Phys. 2020, 127, 175905 10.1063/5.0004757.

